# Supplementary material for: Sensory prediction errors in the human midbrain signal identity violations independent of perceptual distance
Source: eLife. 2019 Apr 5;8:e43962. doi: 10.7554/eLife.43962 (PMC6450666; doi:10.7554/eLife.43962)
Supplement: Supplementary file 1. — Subject-wise parameters for learning rate and choice stochasticity. [file elife-43962-supp1.docx]

**Supplementary File 1: Estimated learning rates and choice stochasticity parameters from reinforcement learning model**

| **Subject** | **Learning rate (alpha)** | **Choice stochasticity (c)** |
| --- | --- | --- |
| 1 | 0.913385 | 0.626913 |
| 2 | 0.945993 | 1.306151 |
| 3 | 0.775926 | 1.073381 |
| 4 | 0.917753 | 0.891103 |
| 5 | 0.936214 | 0.889632 |
| 6 | 0.902333 | 1.358498 |
| 7 | 0.921066 | 1.606549 |
| 8 | 0.872053 | 1.490146 |
| 9 | 0.949318 | 1.155831 |
| 10 | 0.950421 | 1.196337 |
| 11 | 0.943329 | 1.29537 |
| 12 | 0.78739 | 0.689546 |
| 13 | 0.926611 | 0.74277 |
| 14 | 0.952646 | 1.547482 |
| 15 | 0.462899 | 0.753503 |
| 16 | 0.91661 | 1.153186 |
| 17 | 0.82807 | 0.796846 |
| 18 | 0.887772 | 1.26558 |
| 19 | 0.782098 | 1.339287 |
